# Effects of Organic Acidic Products from Discharge-Induced Decomposition of the FRP Matrix on ECR Glass Fibers in Composite Insulators

**DOI:** 10.3390/polym17111540

**Published:** 2025-05-31

**Authors:** Dandan Zhang, Zhiyu Wan, Kexin Shi, Ming Lu, Chao Gao

**Affiliations:** 1 School of Electrical and Electronic Engineering, Huazhong University of Science and Technology, Wuhan 430074, China; wanzhiyu@outlook.com (Z.W.); z5322494@zmail.unsw.edu.au (K.S.); blingblinghello@outlook.com (C.G.); 2 Key Laboratory of Pulsed Power Technology, Huazhong University of Science and Technology, Ministry of Education, Wuhan 430074, China; 3 State Key Laboratory of Advanced Electromagnetic Technology, Huazhong University of Science and Technology, Wuhan 430074, China; 4 State Grid Henan Electric Power Corporation Electric Power Research Institute, Zhengzhou 450052, China; henanluming@foxmail.com

**Keywords:** composite insulator, fiber reinforced polymers, decay-like deterioration, anhydride-cured bisphenol A epoxy resin, ECR glass fiber

## Abstract

This study investigates the degradation mechanisms of fiber-reinforced polymer (FRP) matrices in composite insulators under partial discharge (PD) conditions. The degradation products may further cause deterioration of the electrical and chemical resistance (ECR) glass fibers. Using pyrolysis–gas chromatography-mass spectrometry (PY-GC-MS) and high-performance liquid chromatography–tandem mass spectrometry (HPLC-MS-MS), the thermal degradation gas and liquid products of the degraded FRP matrix were analyzed, revealing the presence of organic acids. These acids form when the epoxy resin’s cross-linked bonds break at high temperatures, generating anhydrides that hydrolyze into carboxylic acids in the presence of moisture. The hydrolyzation process is accelerated by hydroxyl radicals produced during PD. The resulting carboxylic acids deteriorate the glass fibers within the FRP matrix by degrading surface coupling agents and reacting with the alkali metal–silica network, leading to the substitution and precipitation of metal ions. Organic acids, particularly carboxylic acids, were found to have a more severe deteriorating effect on glass fibers compared to inorganic acids, with high temperatures exacerbating this process. These findings provide critical insights into the deterioration mechanisms of FRP under operational conditions, offering valuable guidance for optimizing manufacturing processes and enhancing the longevity of composite insulators.

## 1. Introduction

Composite insulators have been widely utilized in power systems as outdoor insulation equipment. Their core structure consists of a core rod made of fiber-reinforced polymer (FRP), which is externally protected by a silicone-based sheath. Early production of composite insulator core rods used E-glass fibers as the FRP framework. From a chemical composition perspective, glass fibers exhibit alkaline characteristics, making them susceptible to erosion by acidic substances, which consequently leads to a decline in their performance [1].

Specifically, E-glass fibers are prone to degradation in nitric acid environments, making nitric acid a primary cause of brittle fracture in such core rods [2,3]. Some scholars have explored the corrosion mechanisms of E-glass fibers by inorganic acids like nitric acid. Qiu’s research revealed that the degradation process of E-glass fibers in inorganic acids mainly involves the leaching of calcium and aluminum ions. This process is not only influenced by the concentration of H^+^ but also closely related to the types of anions in the acidic solution [4]. Further analysis indicates that the dissolution of boron is a significant factor in the structural damage of the fibers. These findings highlight the critical role of inorganic acids in the corrosion of E-glass fibers.

To address the above issues, new electrical and chemical resistance (ECR) glass fibers have been gradually adopted in the manufacturing of composite insulator core rods. Compared to traditional E-glass fibers, ECR glass fibers significantly enhance acid resistance, particularly in nitric acid environments, by reducing the content of boron oxide (B_2_O_3_), increasing the density of the SiO_2_ network, and raising the proportions of metal oxides such as CaO, Al_2_O_3_, and MgO [1,5,6,7,8]. Furthermore, improvements in the crimping process at the ends of composite insulators have significantly reduced the likelihood of external nitric acid penetrating the FRP core rod and contacting the glass fibers [5]. These technological advancements have effectively mitigated the risk of brittle fracture in composite insulators.

**Figure 1:**
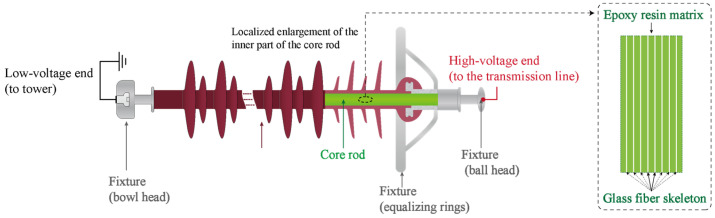
General structure of the composite insulator that includes FRP.

Currently, the matrix material of the FRP core rod is composed of bisphenol A epoxy resin cured with anhydride, while the reinforcing framework is made of ECR glass fibers, as shown in Figure 1. This structure provides high electrical impedance and excellent mechanical strength [5,9,10,11]. However, in recent years, decay-like deterioration and fracture failures caused by partial discharge have become increasingly prevalent. The primary characteristics of decay-like deterioration include significant degradation of the epoxy resin matrix and extensive fracture of glass fibers, particularly with uneven and disordered fiber fracture surfaces. As decay-like deterioration progresses, the FRP core rod eventually fractures, leading to severe consequences such as transmission line tripping.

**Figure 2:**
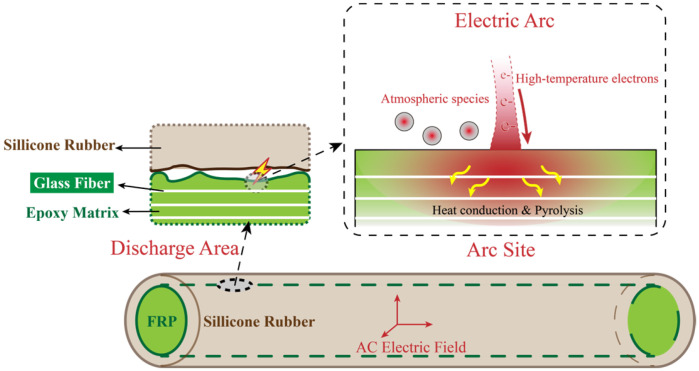
Microscopic process of a single partial discharge: electric arc contacts matrix, creating an instantaneous thermal effect, with atmospheric active species participating in surface reactions.

Current research indicates that partial discharge is the primary cause of decay-like deterioration in composite insulators [12]. As shown in Figure 2, partial discharge can instantaneously generate temperatures as high as 1000 °C within an extremely small volume (approximately 5 × 10^−11^ cm^3^) [13], significantly affecting the thermal properties of the surrounding material surfaces and adjacent areas, potentially leading to material degradation. Additionally, partial discharge can generate reactive species such as free radicals [14]. These reactive species may undergo chemical reactions with the material surface, further accelerating the degradation process, particularly under the synergistic effects of moisture and other atmospheric species.

In FRP composites, the epoxy resin matrix, as a carbon-based polymer, is more susceptible to direct degradation under the influence of partial discharge [15,16,17,18,19]. In contrast, the glass fiber skeleton, primarily composed of silicon–oxygen (Si-O) bonds, exhibits higher chemical stability as an inorganic material and is theoretically less affected by partial discharge. Furthermore, the glass fiber is designed to withstand mechanical loads during power line operation. During actual operation, the overall performance decline and eventual decay-like deterioration of FRP cases often reveal fractures in the glass fiber skeleton, indicating that these fractures are a critical characteristic and factor contributing to the decay-like deterioration of FRP [9,10]. Lu et al. examined 22 composite insulators exhibiting decay-like deterioration, all of which showed evidence of fractured glass fibers [20]. Therefore, a thorough investigation into the fracture mechanisms of the glass fiber skeleton in FRP composite insulators is essential for understanding the microscopic processes underlying decay-like deterioration.

Wan investigated the pyrolysis mechanism of anhydride-cured epoxy resin in composite insulators, preliminarily revealing the formation process of acidic products during pyrolysis [21]. R. Schifani conducted dielectric barrier discharge experiments using epoxy resin containing quartz fillers, exploring the synergistic effects of discharge thermal effects and reactive species in the atmosphere [22]. C. Hudon designed a dielectric barrier discharge experiment in a confined space using plate–plate electrodes under AC voltage, combined with ion chromatography (IC) and X-ray diffraction techniques, suggesting the possible formation of acidic droplets on the surface of epoxy resin [23,24,25]. These studies indicate that FRP epoxy resin matrix materials can indeed generate acidic products under discharge conditions. However, for anhydride-cured epoxy resins widely used in composite insulators, the specific types and formation pathways of acidic products generated during discharge due to thermal effects and atmospheric species involvement still lack systematic research and in-depth analysis.

In addition, although it is widely acknowledged that, at the macroscopic level, discharge corrosion in humid environments is the primary cause of the eventual fracture of acid-resistant core rods, the specific microscopic mechanisms underlying the fracture of glass fibers during this deterioration process remain unclear [5,9,10,11,26,27,28]. Li’s research, through the analysis of boron-containing (B_2_O_3_) E-glass fibers and boron-free ECR glass fibers in sulfuric acid, confirmed the negative impact of boron on acid resistance [8]. Concurrently, Xie’s study, by comparing the acid resistance of E-glass fibers and ECR glass fibers in various inorganic acids, further validated the superior acid resistance of ECR glass fibers [7]. This indicates that the deterioration effect of inorganic acids on the widely used ECR glass fibers is relatively limited. Therefore, it is hypothesized that, beyond inorganic acids, other potential factors contribute to their decay-like deterioration and even fracture.

To investigate the mechanism behind the decay-like deterioration of FRP matrix in composite insulators, characterized by FRP matrix degradation and skeleton fracture, despite the use of acid-resistant ECR glass fibers as the reinforcement, this study first employs PY-GC-MS to simulate the thermal effects of partial discharge on the surrounding FRP matrix and adjacent regions, followed by the detection and analysis of thermal degradation gas products. Subsequently, a partial discharge simulation experiment under the synergistic effects of atmospheric species is conducted to explore the impact of radicals and other byproducts generated during partial discharge, as well as the synergistic effects of atmospheric species, on the surrounding FRP matrix. HPLC-MS-MS is utilized to detect and analyze the liquid products generated in the experiment. Following this, the acidity of the products is evaluated based on their molecular structure and acid dissociation constant (pKa), and acidic substances potentially corrosive to glass fibers are identified. Further, considering the operating temperatures of outdoor electrical equipment, the effects of typical organic acids on glass fibers at different temperatures and their corrosion mechanisms are analyzed, and the results are compared with the corrosion effects of inorganic acids such as nitric acid. The findings of this study can provide a theoretical basis for optimizing the manufacturing process of FRP in composite insulators, thereby enhancing the performance and lifespan of electrical equipment, and achieving comprehensive detection of epoxy resin matrix pyrolysis products.

## 2. Materials and Methods

### 2.1. The Structure of the Epoxy Resin Matrix and the Glass Fiber Skeletal Material

**Figure 3:**
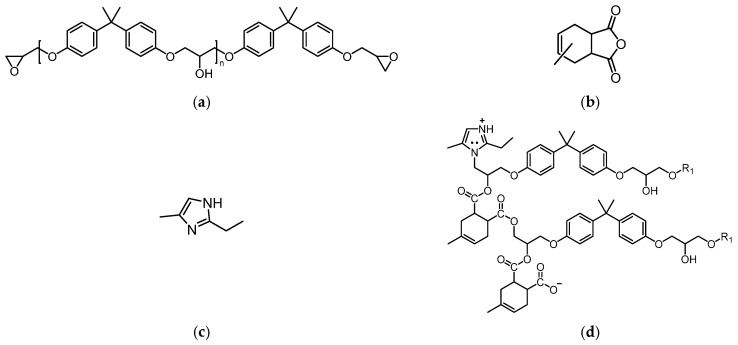
Molecular structures of key materials used in synthesizing the epoxy resin for this study and the resulting cross-linked network, they should be listed as: (**a**) DGEBA epoxy resin; (**b**) MeTHPA; (**c**) 2-Ethyl-4-methylimidazole; (**d**) Crosslinked network after curing (Reproduced with permission from [21], Elsevier, 2024).

Anhydride-cured epoxy resins are widely used as the FRP matrix material for composite insulator core rods due to their excellent dielectric and mechanical properties. Figure 3a–c illustrate the molecular structures involved in the synthesis of the epoxy resin, with bisphenol A diglyceryl ether (DGEBA) as the primary component, having an equivalent weight range of 183–190 g/mol. The recipe of materials utilized in this study is derived from what has been consulted from the manufacturer to enhance the real case modification. Methyl tetrahydrophthalic anhydride (MeTHPA) serves as the curing agent, with an anhydride group content of no less than 40.5%, and its usage is approximately 85% of that of DGEBA. A small amount of 2-ethyl-4-methylimidazole is added as an accelerator, with a dosage of 2–7 parts per hundred parts of resin (phr) [29]. After mixing the materials, curing is carried out at 120 °C for 4 h, followed by natural cooling to room temperature. During this process, 2-ethyl-4-methylimidazole catalyzes the chain-growth polymerization reaction between DGEBA and MeTHPA, forming a cross-linked network as shown in Figure 3d [30]. Here, DGEBA constitutes the main chain, while MeTHPA reacts with the epoxy groups of DGEBA to form cross-linking nodes [21].

**Figure 4:**
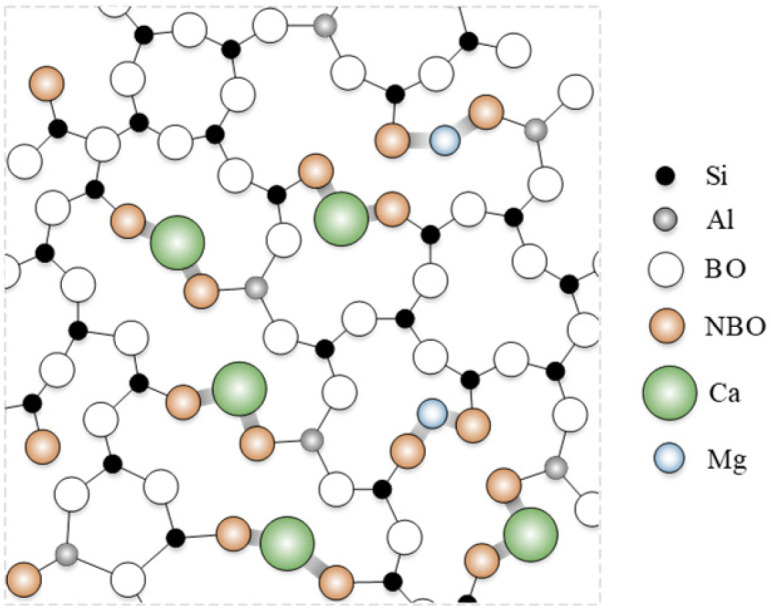
The space structure schematic of ECR glass fiber.

In the core rod of composite insulators, the glass fiber skeleton accounts for approximately 80% of the total weight and 65% of the total volume. As shown in Figure 4, the SiO_4_^4−^ tetrahedra form the primary network skeleton of ECR glass fibers, providing rigid structural support. The introduction of AlO_4_^5−^ tetrahedra embeds them into the Si-O-Al network structure, reducing the number of non-bridging oxygens (NBO) while increasing the content of bridging oxygens (BO), thereby optimizing the network structure of the glass fibers. Additionally, Ca^2+^ and Mg^2+^, as key network-modifying ions, not only fill the network gaps and increase network density but also enhance network stability by compensating for the negative charge of AlO_4_^5−^, ultimately contributing to the improvement of the elastic modulus of the glass fibers. Although ECR glass fibers eliminate a significant amount of easily degradable BO_3_^3−^ planes introduced by boron, weak points still exist in the system—metal oxides exhibit alkalinity and are theoretically susceptible to erosion by acidic substances, leading to their departure from the network structure. Therefore, this study will next focus on investigating the effects of acidic substances on glass fibers and their underlying mechanisms.

### 2.2. Experimental Methods for Discharge-Induced Deterioration

#### 2.2.1. Simulation Experiment on Thermal Effects of Partial Discharge

**Figure 5:**
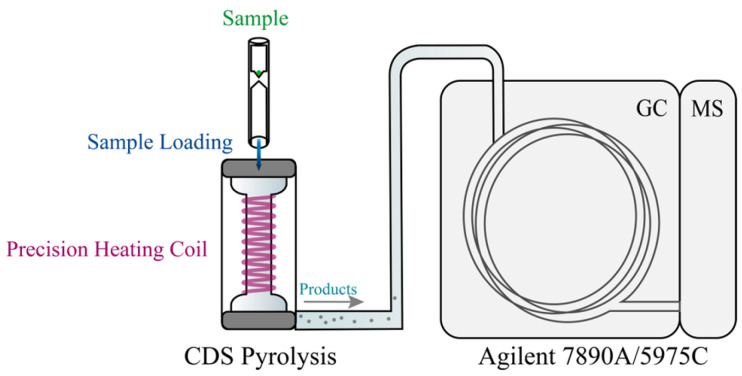
Schematic diagram of PY-GC-MS analysis of decomposition products of epoxy matrix (reproduced with permission from [21], Elsevier, 2024).

Considering the extremely high temperatures generated during partial discharge and its adiabatic nature, the thermal effects on the surrounding FRP matrix and adjacent regions cannot be overlooked. To simulate this physical phenomenon, PY-GC-MS was employed to replicate the intense pyrolysis process of epoxy/anhydride thermosetting materials under instantaneous high temperatures. As shown in Figure 5, the experimental setup included a CDS Analytical 6150 pyrolyzer (from CDS Analytical, Inc., Oxford, PA, USA) and an Agilent 7890A/5975C GC-MS system (from Agilent Technologies, Inc., Santa Clara, CA, USA). Additionally, the varying thermal effects at different locations around the discharge area on the FRP matrix and adjacent regions were translated into the degradation of epoxy resin at different temperatures, with a defined temperature range of 200–1000 °C.

#### 2.2.2. Discharge Simulation Experiments Considering Atmospheric Species and Thermal Effects

**Figure 6:**
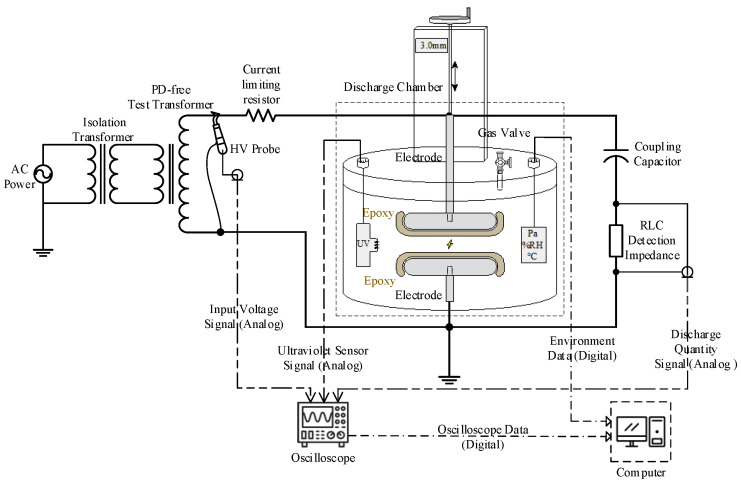
Discharge experiment chamber, high voltage circuit, and detection system (Reproduced with permission from [32], Springer, 2025).

To further investigate the effects of partial discharge on the surface of the FRP matrix, this study comprehensively considers the combined effects of thermal effects, generated free radicals, and synergistic interactions with atmospheric species. An enclosed chamber discharge experimental platform, as shown in Figure 6, was constructed. The experiment utilized upper and lower electrode disks, which tightly encapsulated pretreated epoxy resin samples. By applying an external high voltage, discharge was induced in the air gap between the samples to simulate the partial discharge effects within air gap defects. Prior to the experiment, the chamber atmosphere was set to one atmospheric pressure with a gas composition of 78% nitrogen and 22% oxygen. A glass dish containing deionized water was placed inside the chamber to maintain 100% relative humidity (RH).

The experimental power supply utilized an isolation transformer and a partial discharge-free test transformer to provide a high voltage of 15 kV RMS. The output voltage was precisely controlled using a high-voltage probe. The discharge quantity was measured in accordance with the IEC 60270:2000 standard [31]. The discharge detection circuit consisted of a coupling capacitor and an RLC detection impedance, which was calibrated using a pulse generator prior to the experiment. The discharge quantity was calculated by monitoring the voltage amplitude output from the RLC detection impedance and multiplying it by the calibration factor. To minimize the impact of environmental electromagnetic interference, an ultraviolet (UV) detector was introduced to capture discharge light pulses, which were then compared with the voltage pulses from the RLC detection impedance to ensure the accuracy of the discharge phase and relative pulse intensity. The discharge pulse signals were recorded using an oscilloscope and an upper computer. During the experiment, the discharge quantity was controlled within the range of 500–1500 pC, with a discharge repetition rate of approximately 5 kHz, and the experiment lasted for 5 h.

After the experiment, the liquid products of epoxy resin under discharge conditions in the aforementioned sealed chamber were analyzed using a high-performance liquid chromatography–tandem mass spectrometry (LC-MS/MS) system. The liquid chromatography system employed was the Thermo Fisher UltiMate 3000 HPLC (from Waltham, MA, USA), coupled with a Thermo Fisher Q Exactive quadrupole-Orbitrap hybrid mass spectrometer (from Waltham, MA, USA) equipped with a high-efficiency electrospray ionization source (HESI). The system was capable of switching between positive and negative ionization modes at a rate of less than 1 s per cycle. This study utilized the data-dependent acquisition (DDA) mode, where high-intensity precursor ions detected in the primary MS scan were subjected to MS2 scanning and fragmented via high-energy collision dissociation (HCD) mode with a collision energy of 40 eV. The fragment ions were analyzed by the Orbitrap mass spectrometer to obtain high-resolution mass spectrometry data, with an *m*/*z* scanning range of 50–750.

### 2.3. The Experiment on the Degradation of Glass Fibers Under Acidic and Thermal Conditions

To investigate the mechanism behind the fracture of composite insulators with FRP matrices reinforced by acid-resistant ECR glass fibers, this study builds upon existing research on the effects of acids on glass fibers. Additionally, it considers the potential high-temperature conditions caused by factors such as partial discharge, dielectric loss, leakage currents, and operational high-temperature environments in composite insulators. An acid–thermal synergistic degradation experimental platform was designed and constructed, capable of simultaneously creating high-temperature and acidic conditions. By incorporating a condenser tube, the platform effectively suppresses the volatilization of acid during heating.

Prior to the experiments, to simulate the high-temperature environment that insulators may experience and to evaluate the effects of acidic solutions, this study selected 25 °C and 80 °C as the degradation temperatures based on surface temperature data from insulators exhibiting abnormal heating during actual operation. In conjunction with the findings on the discharge decomposition products of the FRP matrix in composite insulators discussed later in this study, typical organic acids (e.g., oxalic acid) and inorganic acids (e.g., nitric acid) were chosen to simulate different acidic environments. The potential effects of acidic conditions on glass fibers were also investigated. Since oxalic acid is a dibasic acid and nitric acid is a monobasic acid, both solutions were prepared to have the same effective H^+^ ion concentration. Specifically, 0.5 mol/L oxalic acid and 1 mol/L nitric acid were used for comparative experiments. The experimental duration was set to 12 days. For ease of expression, the experimental conditions are denoted as temperature-concentration-acid type-degradation time, for example: 25 °C-0.5 mol/L oxalic acid-12 d.

To compare the mechanical properties of ECR glass fiber samples used in composite insulators before and after degradation, the study employed a tensile testing machine to conduct stress tests. The tensile fracture stress (*σ*) and elastic modulus (*E*) before and after degradation were calculated using Equations (1)–(3).

(1) $$\sigma =\frac{F}{S}$$

(2) $$\varepsilon =\frac{\Delta L}{L_{0}}$$

(3) $$E=\frac{\sigma }{\varepsilon }$$

In the equation, F represents the applied stress measured by the tensile testing machine, S denotes the cross-sectional area of the test specimen, σ is the tensile fracture stress, ΔL indicates the change in the length of the specimen, $L_{0}$ is the original length of the specimen, ε stands for strain, and E represents the elastic modulus.

This study employed Fourier Transform Infrared Spectroscopy (Thermo Scientific Nicolet iS50R FTIR, from Thermo Fisher Scientific Inc., Waltham, MA, USA) and X-ray Energy Dispersive Spectroscopy (FEI Sirion 200 EDS, from FEI Company, Hillsboro, OR, USA) to detect changes in chemical elements and functional groups of the samples before and after the experiments. Additionally, X-ray Diffraction (Malvern Panalytical X’Pert^3^ Powder XRD, from Malvern Panalytical, Almelo, The Netherlands) was utilized to examine the crystallization of the solution post-experiment. More specifically, the utilized Nicolet iS50R spectrometer was equipped with a diamond Attenuated Total Reflection (ATR) accessory, and the measurement parameters were set to 45° angle of incidence and single reflection mode, with a wave number scanning range of 4000–400 cm^−1^ and a resolution of 4 cm^−1^. XRD was performed using a copper target (Cu, 40 kV, 40 mA) as the X-ray source for the characterization of the physical phases in a scanning range of 5° to 90° with a step size of 0.013°. By integrating the results from these analyses, the mechanisms by which acidic solutions and temperature affect glass fibers were systematically investigated.

## 3. Results and Discussion

### 3.1. Analysis of Acidic Byproducts from Discharge

#### 3.1.1. Acidic Products from Thermal Effects of Discharge

**Figure 7:**
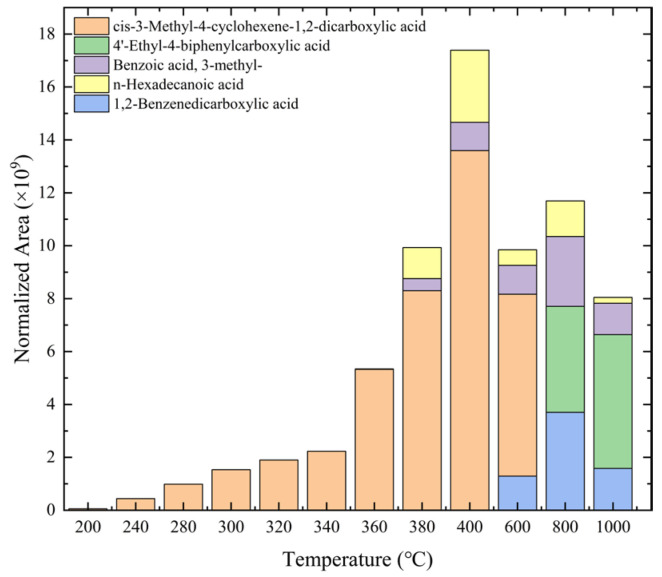
Variation in types and quantities of identified acidic products with pyrolysis temperature.

Table 1 and Figure 7 present the results of various acidic products derived from epoxy resin samples analyzed using PY-GC-MS. The peak areas of the products from different experimental groups were normalized according to the method described in reference [21]. Specifically, Table 1 provides the retention time (RT), corresponding names, chemical formulas, chemical structures, molecular weights (Mol. wt.), and pKa values, which indicate the dissociation strength of the products in solution (the acquired chromatograms for Table 1 are provided in Figure S1 of the Supplementary Materials). Figure 7 illustrates the normalized areas of different products over time.

This study primarily focuses on acidic organic compounds with a pKa below 5. As shown in Table 1 and Figure 7, cis-3-methyl-4-cyclohexene-1,2-dicarboxylic acid becomes a significant organic acid product when the pyrolysis temperature exceeds 240 °C. At 400 °C, the yield of this product reaches its peak, which is 31 times higher than that at 240 °C. However, as the pyrolysis temperature further increases, this organic acid gradually decomposes. At 600 °C, 800 °C, and 1000 °C, its yield decreases by 40%, 89%, and 97%, respectively, compared to that at 400 °C. When the temperature rises above 600 °C, 1,2-benzenedicarboxylic acid begins to form, reaching approximately three times the yield at 600 °C by 800 °C. However, at 1000 °C, its yield is only 43% of that at 800 °C.

**Figure 8:**
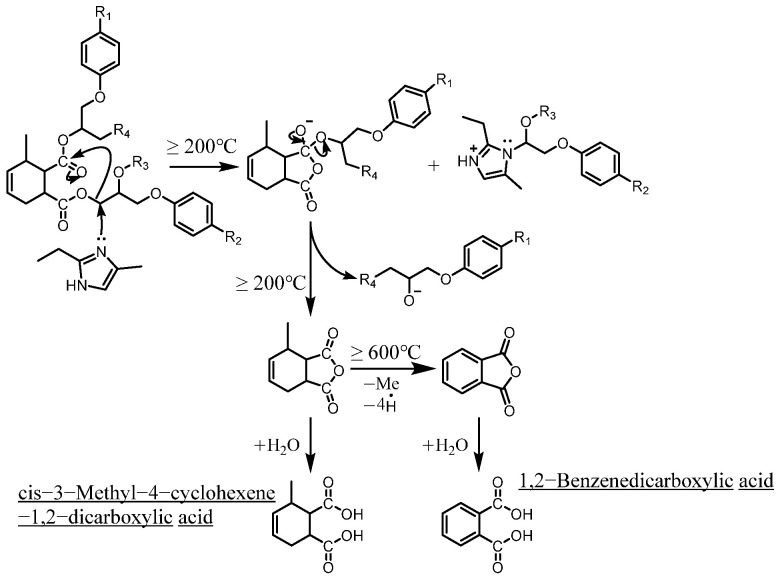
Thermal decomposition pathways for the formation of identified dicarboxylic acids (reproduced with permission from [21], Elsevier, 2024).

As mentioned in reference [14], these dicarboxylic acids primarily originate from the cleavage of anhydride crosslinking points in the cured epoxy resin network. Once the ceiling temperature is reached, anhydrides are prone to depolymerization even at relatively low pyrolysis temperatures (200 °C), subsequently hydrolyzing to form organic acids (e.g., dicarboxylic acids). These dicarboxylic acids may further induce the leaching of metal ions from the glass fibers in composite insulators, leading to a decline in their mechanical properties. The specific thermal decomposition pathways are illustrated in Figure 8 [21].

#### 3.1.2. Thermal Effects of Acidic Products Under the Influence of Atmospheric Species

**Figure 9:**
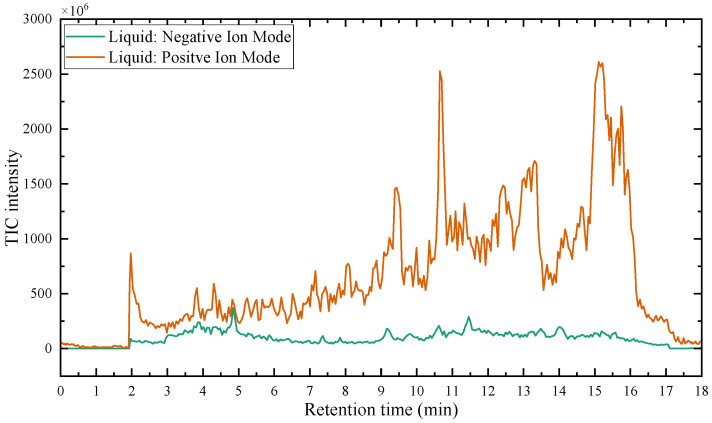
The TIC curve of the liquid epoxy resin products was detected using LC-MS/MS.

Figure 9 presents the total ion chromatogram (TIC) curves obtained by LC-MS/MS in both negative and positive ion modes, showing the variation with RT. The *x*-axis represents RT, and the *y*-axis represents the intensity. By analyzing the primary and secondary mass spectrometry data in both positive and negative ion modes, acidic degradation products of the epoxy resin liquid were identified. Substances with peak areas exceeding 1.0 × 10^8^ were selected and listed in Table 2. The extracted-ion chromatogram (XIC) values for certain compounds, derived from Figure 9 and displayed in Table 2, are illustrated in Figure S2 of the Supplementary Materials.

The results in Table 2 indicate that the thermal effects of epoxy resin under the influence of atmospheric species produce a variety of acidic products, with a greater diversity of organic acid products compared to the results in Table 1. This suggests that partial discharge generates a wider range of acidic products on the surface of the epoxy resin. This phenomenon explains why the deterioration of composite insulators in practical applications initially occurs at the interface. A significant amount of carboxylic acid primarily originates from the breakdown of cross-linking points, forming anhydrides, which further undergo hydrolysis in the presence of moisture. Compared to the effects of thermal action alone, environmental moisture and hydroxyl radicals generated by humid gas discharge accelerate this process. According to previously published experimental data, the lifespan of hydroxyl radicals, which can accelerate the hydrolyzation process during PD, ranges from nanoseconds (10^−9^ s) to milliseconds (10^−3^ s) under such similar condition (gas-phase environment), and the corresponding concentration is usually in the range of 10^12^–10^14^ cm^−3^ [33,34,35,36]. Additionally, the formation of nitric acid was detected, attributed to the reaction of nitrogen oxides (NO_x_) produced from the interaction of nitrogen (N_2_) and oxygen (O_2_) in the air during discharge, with water. Therefore, the acidic products resulting from the thermal effects of epoxy resin under the influence of atmospheric species are more diverse than those produced under thermal effects alone.

Based on the calculated peak areas of acidic products in Table 2, the total peak area of acidic products is 33.20 × 10^8^, with organic acids accounting for 94.58%, which is 17.45 times that of inorganic acids. This result indicates that, under the thermal effects influenced by atmospheric species, epoxy resin primarily generates strongly acidic organic acids. Therefore, focusing solely on the impact of inorganic acids on the material is insufficient. The organic acids produced after partial discharge may affect the performance of ECR composite insulators and further promote their decay-like deterioration. This potential mechanism warrants in-depth investigation.

### 3.2. The Impact of Organic Acids on ECR Glass Fibers

Although ECR glass fibers exhibit strong acid resistance, fractures in the FRP core rods of composite insulators still occur during practical applications. Research in Section 3.1 indicates that the thermal effects of discharge, either independently or in synergy with atmospheric species, can generate acidic products such as carboxylic acids. However, current studies predominantly focus on inorganic acids, leaving a gap in understanding the impact of organic acids on ECR glass fibers. Therefore, this study selects oxalic acid, a typical dicarboxylic acid, as a low-molecular-weight organic acid corrosive agent. Additionally, considering that transient high temperatures induced by partial discharge and dielectric loss may accelerate the aging of ECR glass fibers, this study systematically investigates the deterioration behavior of ECR glass fibers under acidic and high-temperature conditions. Furthermore, the oxalic acid solution utilized in this study is deemed stable throughout the experiment, based on three considerations: oxalic acid, as a polar molecule, exhibits non-volatility; oxalic acid crystals will reform an acidic solution upon interaction with environmental water molecules; and partial discharges will persist once initiated.

**Figure 10:**
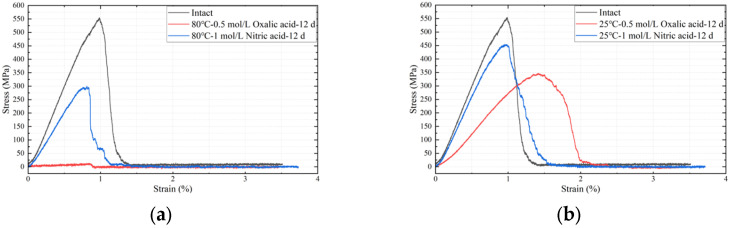
Stress–strain curves of glass fibers after deterioration under different acid–heat conditions, they should be listed as: (**a**) varied acids at 80 °C after 12 d; (**b**) varied acids at 25 °C after 12 d.

Figure 10 illustrates the stress–strain curves of glass fibers under different acid solutions and temperature conditions. The results indicate that the stress–strain curves of ECR glass fibers after exposure to acid and heat are lower than those of untreated specimens unaffected by acid and high temperature. At the same experimental temperature, the stress–strain curves of the oxalic acid group are lower than those of the nitric acid group. When the acid type is the same, the stress–strain curves of the high-temperature (80 °C) experimental group are lower.

Table 3 presents the average tensile fracture stress and elastic modulus of three parallel experiments under each parameter, and their relative decline compared to the intact state of glass fibers under different acid solutions and temperature conditions.

According to Table 3, under the same temperature conditions, the impact of oxalic acid on the mechanical properties of glass fibers is significantly greater than that of nitric acid. For instance, at a solution temperature of 25 °C, the tensile fracture stress of glass fibers in the nitric acid experimental group decreased by only 13.03%, while the oxalic acid experimental group showed a decrease of 36.46%, approximately 2.8 times that of the nitric acid group. When the solution temperature increased to 80 °C, the reduction in the nitric acid group rose to 41.17%, whereas the oxalic acid group experienced a decline of 96.12%, about 2.3 times that of the nitric acid group. Overall, at the same temperature, the reduction in tensile fracture stress in the oxalic acid experimental group was roughly 2–3 times that of the nitric acid group, with a similar 2–3 times ratio observed in the reduction in elastic modulus between the two groups. Generally, the relative standard deviation (RSD) under each parameter is within 5.26%.

The data in Table 3 also indicate that an increase in temperature leads to a decline in the mechanical properties of ECR glass fibers, with this phenomenon being more pronounced in the oxalic acid experimental group. Taking the reduction in elastic modulus as an example, when the temperature increased from 25 °C to 80 °C, the value in the oxalic acid experimental group rose from 37.65% to approximately 94.22%, representing a total change of about 60%. In contrast, the results for the nitric acid experimental group showed a relatively gradual change of about 25% with temperature variation.

According to Equation (4) of Arrhenius’ law, the rate constant k of a chemical reaction increases with the rise in absolute temperature *T*.

(4) $$k=Ae^{-\frac{E_{a}}{RT}}$$

In the equation, A represents the pre-exponential factor, $E_{a}$ denotes the activation energy, R is the gas constant, approximately 8.314 J/(mol·K), and e is the base of the natural logarithm, approximately 2.718. Equation (4) indicates that higher temperatures accelerate the chemical reactions between the acid and the glass fibers, leading to a rapid decline in mechanical properties. This explains the experimental observation that the mechanical properties of the samples decrease more significantly under elevated temperature conditions when exposed to the same acid. From the perspective of the materials involved in the experiment, the increase in temperature enhances the diffusion rate of ions and molecules in the acid, intensifying the erosion of the glass fibers’ internal structure by the acid, thereby causing a swift deterioration in the mechanical properties of the samples. Furthermore, the rise in temperature also affects the thermodynamic stability of the glass fibers, making them more susceptible to chemical corrosion under the same acidic conditions.

**Figure 11:**
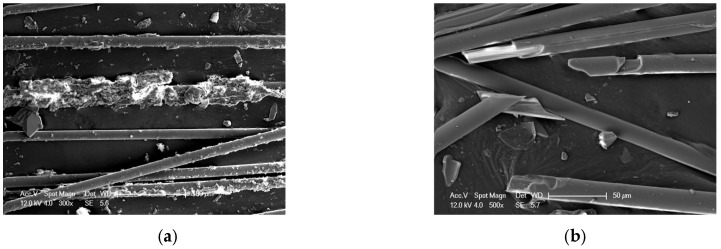
Microscopic morphology of glass fibers under the influence of acid–heat: (**a**) 80 °C-1.0 mol/L nitric acid-12 d, (**b**) 80 °C-0.5 mol/L oxalic acid-12 d.

Under the influence of acid and heating, the surface of glass fibers exhibits damage or even fracture. As shown in Figure 11, ECR glass fibers demonstrate surface damage when exposed to 80 °C-1.0 mol/L Nitric acid for 12 days, while significant fracture is observed in samples treated with 80 °C-0.5 mol/L oxalic acid for the same duration. This phenomenon suggests a potential significant decline in the mechanical properties of the glass fibers under these conditions.

### 3.3. The Erosion Mechanism of Organic Acids on ECR Glass Fibers

The results in Section 3.2 indicate that the mechanical properties of ECR glass fiber samples significantly decline under the influence of acidic solutions, with organic acids (e.g., oxalic acid) exerting a greater impact on their performance compared to inorganic acids (e.g., nitric acid). Heating conditions further accelerate this degradation process. To clarify the mechanisms by which the type of acid and temperature affect glass fibers, EDS and FTIR analyses were conducted on the ECR glass fiber samples before and after the experiments. Both measurements focus on the phenomena on average at the surface of samples.

#### 3.3.1. Chemical Element Changes

**Figure 12:**
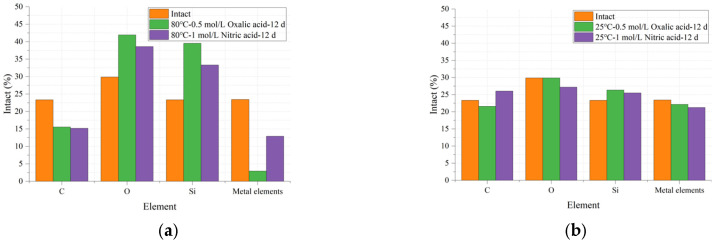
Comparison of the elemental content of glass fibers in their original state and after deterioration under various conditions: (**a**) varied acids at 80 °C after 12 d; (**b**) varied acids at 25 °C after 12 d.

The EDS results are shown in Figure 12, where the vertical axis shows the relative content percentage of each element and the horizontal axis shows the types of elements. Table 4 shows the reduction in each element under different conditions compared to their intact states. Generally, three parallel measurements were carried out, and the relevant RSD value for each element content is within 6.23%.

The results in Figure 12 indicate that the carbon (C) content in the glass fiber samples decreased after the acid–heat experiments, and this decline was exacerbated with increasing temperature. As shown in Table 4, for the oxalic acid experimental group, the C content decreased by 7.45% at 25 °C and by 34.99% at 80 °C, representing a change of approximately 37%. A similar trend was observed in the nitric acid (HNO_3_) experimental group.

The primary source of C element is the coupling agent in the glass fiber sample. The reduction in the content of this element indicates that the coupling agent was damaged during the experiment. The failure of the coupling agent exposes the ECR glass fiber, making it susceptible to acid erosion, which leads to a decline in the mechanical properties of the sample. According to the data in Table 3, for the same acid solution experimental group, the tensile fracture stress and elastic modulus of the ECR glass fiber indeed decrease with increasing temperature. In practical applications, composite insulators with degraded mechanical properties may ultimately undergo decay-like deterioration or even fracture under external forces.

In addition to carbon (C) elements, the content of metallic elements (Ca, Mg, etc.) in Figure 12 also showed a declining trend after the acid–heat experiments, and temperature significantly accelerated this trend. The results in Table 4 indicate that under conditions of 25 °C and 80 °C, the decrease in metallic element content in the oxalic acid experimental group was 5.46% and 87.45%, respectively, representing a change of approximately 82%. In contrast, the results for the nitric acid group increased from 9.26% to 44.94%, representing a change of approximately 55%. This phenomenon also demonstrates that, under the same temperature variation, oxalic acid has a greater impact on the metallic element content in ECR glass fibers compared to nitric acid. This observation aligns with the effects of acid solutions on the mechanical properties of ECR glass fibers, as shown in Table 3.

The underlying reason is that H^+^ in the acid solution undergoes ion exchange reactions with the alkali metal–silicon network of the glass fibers, as shown in Equation(5), causing the metal elements in the sample to leach out from the glass structure, resulting in a decrease in their content. The leached Ca^2+^ reacts with oxalate ions (C_2_O_4_^2−^) to form complexation reactions, as shown in Equations (6) and (7), generating sparingly soluble calcium oxalate monohydrate (CaC_2_O_4_·H_2_O, with a solubility of approximately 0.0067 g/L at 25 °C). Meanwhile, nitrate ions (NO^3−^) form highly soluble calcium nitrate (Ca(NO_3_)_2_, with a solubility of 1212 g/L at 25 °C), which exists in the solution in ionic form, as shown in Equations (8) and (9). The precipitation of calcium oxalate significantly reduces the concentration of Ca^2+^ in the solution. According to Le Chatelier’s principle, this process drives more Ca^2+^ to leach out from the glass fibers, forming a self-enhancing corrosion cycle. This explains why the metal element content in the nitric acid group decreased by 44.94% at 80 °C, while the oxalic acid group showed a decrease of twice as much, at 87.45%. Additionally, the reactions described in Equations(5)–(9) lead to a reduction in the metal element content in the sample, which accounts for the observed decline in metal element content after the acid–thermal experiments.

(5) $$[\mathrm{Si}\text{-}O{]}_{2}\text{-}Ca+H^{+}\to 2Si\text{-}\mathrm{OH}+{\mathrm{Ca}}^{2+}$$

(6) $${\mathrm{Ca}}^{2+}+C_{2}O_{4}^{2-}\to \mathrm{Ca}C_{2}O_{4}$$

(7) $$\mathrm{Ca}C_{2}O_{4}+H_{2}O\to \mathrm{Ca}C_{2}O_{4}\cdot H_{2}O$$

(8) $${\mathrm{Ca}}^{2+}+2NO_{3}^{-}\to \mathrm{Ca}{(NO_{3})}_{2}$$

(9) $$\mathrm{Ca}{(NO_{3})}_{2}+{4H}_{2}O\to \mathrm{Ca}{(NO_{3})}_{2}\cdot 4H_{2}O$$

In Figure 12, the content of O and Si elements shows an increasing trend with rising temperature. Specifically, the O element content in the oxalic acid group increases from 0.07% to 40.37%, while the Si element content rises from 12.85% to 69.38%. As Si is the primary element in ECR glass fibers, its content variation serves as a crucial indicator of structural changes in composite insulators. The 80 °C-0.5 mol/L oxalic acid-12 d experimental group exhibits the most significant change in Si content, reaching 69.38%, compared to intact samples. Combined with the data in Table 3, it is evident that the mechanical properties of the 80 °C-0.5 mol/L oxalic acid-12 d experimental group also experience the most pronounced decline.

The increase in the relative content of O and Si elements under the influence of temperature and acid is attributed to the destruction of the coupling agent on the surface of the glass fiber sample, which exposes the O and Si elements originally located in the subsurface layer. This process is accompanied by the aforementioned loss of C and metallic elements. Additionally, in an acidic environment, the metallic elements present in the sample may undergo oxidation reactions, forming oxides or silicates. These reaction products tend to accumulate on the surface of the glass fiber sample, leading to an increase in the relative content of O and Si elements as detected by EDS.

Overall, Figure 12 demonstrates that an increase in temperature exacerbates the decline in the relative content of C and metallic elements in the sample, while promoting the rise in the relative content of O and Si elements. Higher temperatures accelerate the rate of chemical reactions, making the erosion of glass fibers by acidic solutions more pronounced. Additionally, elevated temperatures enhance the solubility of many compounds, further contributing to the observed changes in elemental content as detected by EDS.

#### 3.3.2. Analysis of Crystalline Products

**Figure 13:**
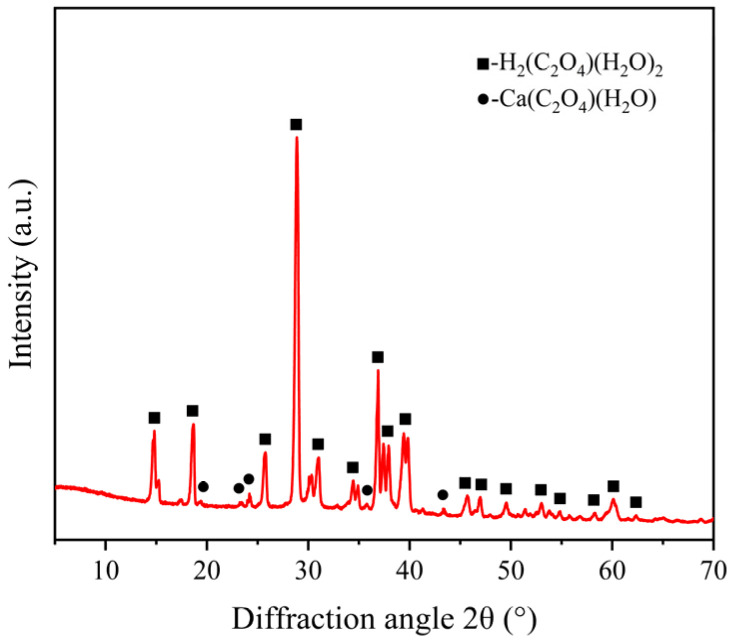
XRD results of crystals formed from the dried solution.

To identify the chemical reaction products of ECR glass fibers under acidic and thermal conditions, the crystals formed after drying the solution were analyzed using XRD. The results are shown in Figure 13, where the horizontal axis represents the diffraction angle and the vertical axis indicates the relative intensity.

Figure 13 indicates that diffraction peaks corresponding to the (−101), (101), (110), (−204), and (−114) crystal planes were detected at diffraction angles (2*θ*) of approximately 14.8°, 18.6°, 28.9°, 36.9°, and 39.4°, respectively. Referring to the standard pattern file PDF#04-009-3739, the crystal structure belongs to the monoclinic system with a space group of P21/n (14), corresponding to oxalic acid dihydrate (H_2_(C_2_O_4_)(H_2_O)_2_). Additionally, diffraction peaks corresponding to the (−102), (121), (040), (−223), and (−144) crystal planes were detected at diffraction angles (2*θ*) of approximately 19.4°, 23.3°, 24.2°, 35.8°, and 43.4°, respectively. Referring to the standard pattern file PDF#04-011-6806, the crystal structure also belongs to the monoclinic system, with a space group of P21/c (14), corresponding to calcium oxalate hydrate (Ca(C_2_O_4_)(H_2_O)).

Through whole-pattern fitting (WPF) and Rietveld refinement calculations, the relative content of the phases in Figure 13 was determined. The sample exhibited 95.0% oxalic acid crystals (H_2_(C_2_O_4_)(H_2_O)_2_) and 5.0% calcium oxalate complex crystals (Ca(C_2_O_4_)(H_2_O)), which are the products of reaction (7). The oxalic acid crystals primarily originated from the crystallization of the dried oxalic acid solution, while the calcium oxalate complex was formed by the reaction of oxalate ions with Ca^2+^ metal ions in the glass fiber framework. The results in Figure 13 confirm the existence of reactions (5)–(7) and validate the correctness of the previously proposed mechanism for the decline in metal element content. The precipitation and crystallization of metallic elements do result in a reduction in their mechanical properties, as evidenced by the changes in mechanical properties in Figure 10.

#### 3.3.3. Chemical Functional Groups Changes

**Figure 14:**
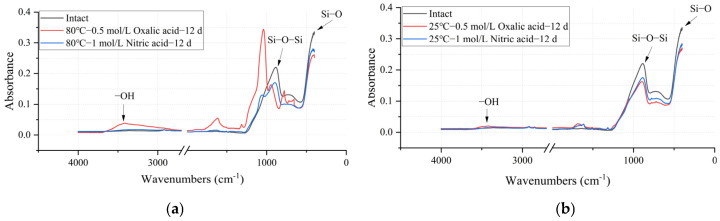
FTIR spectra of intact and deteriorated glass fibers under different acid–heat conditions: (**a**) deteriorated at 25 °C for 12 days with 0.5 mol/L oxalic acid and 1 mol/L nitric acid; (**b**) deteriorated at 80 °C for 12 days with 0.5 mol/L oxalic acid and 1 mol/L nitric acid.

Figure 14 presents the FTIR detection results of the main functional groups of the glass fiber samples under different acid solutions and temperature conditions, with the wavenumber on the horizontal axis and the absorbance on the vertical axis. In this section, the absorbance of the main functional group bands in Figure 14 was integrated to obtain peak area data reflecting the relative content of each functional group. Table 5 shows the variation in peak areas of the main functional groups of the samples after the experiments. Generally, three parallel measurements were carried out, and the relevant RSD value for each principal functional group content is within 4.88%.

As shown in Table 5, the content of Si-O and Si-O-Si groups, which serve as the primary structural framework of the sample, decreased after the acid–heat experiments. This phenomenon indicates that the structure of the glass fiber was damaged. Similarly to the mechanical performance data in Table 3, under the same acid conditions, an increase in temperature exacerbated the structural changes in the ECR glass fiber. Taking the oxalic acid experimental group as an example, when the temperature increased from 25°C to 80°C, the Si-O group content relative to the intact state changed from 83.6% to 45.22%, while the Si-O-Si group content changed from 80.51% to 31.36%. Furthermore, the extent of change in Si-O and Si-O-Si group content with temperature was more pronounced in the oxalic acid group than in the nitric acid group, indicating that the structure of the ECR glass fiber was more significantly affected by oxalic acid than by nitric acid.

In summary, when partial discharge occurs in composite insulators, organic acids are generated in the epoxy resin matrix, which further damages the composition and structure of ECR glass fibers. Since partial discharge is a significant driving factor for the decay-like deterioration of composite insulators, preventing partial discharge or mitigating its deteriorating effects on materials is crucial for extending the service life of equipment. On one hand, it is essential to optimize manufacturing processes and standards before product delivery to eliminate defects that may lead to partial discharge during production and operation. On the other hand, given that sporadic discharge defects may still occur even with optimized processes, it is necessary to enhance the material’s resistance to long-term secondary effects that contribute to decay-like deterioration. Based on the results in Section 3.3, it is recommended to use glass fibers with lower Ca metal content in practical applications to mitigate ion leaching and damage caused by organic acids and high-temperature environments. Furthermore, studies have shown that the destructive effect of organic acids on glass fibers is significantly greater than that of inorganic acids, so efforts should be made to minimize the generation of organic acids inside the core rod. According to Campbell’s research [37], reducing the amount of imidazole catalyst in FRP core rod production, ensuring the stoichiometric ratio of epoxy resin exceeds that of anhydride, and preferentially using tetrahydrophthalic anhydride (THPA) instead of methyltetrahydrophthalic anhydride (MeTHPA) can effectively reduce the volatilization of anhydride, which can further reduce carboxylic acids generated by the hydrolysis of anhydride.

## 4. Conclusions

This research comprehensively analyzed the discharge breakdown products of the FRP matrix in composite insulators utilizing PY-GC-MS and HPLC-MS-MS techniques. The production of organic acids was verified, and the detrimental impacts of these acids on the ECR glass fibers in the FRP core rod material were meticulously examined. The research offers experiment-based results for future study on enhancing the manufacturing process and prolonging the lifespan of composite insulators. The primary conclusions are as follows:

(1) When the polymerization ceiling temperature exceeds the critical threshold, cross-linking points begin to break, generating anhydrides. These anhydrides subsequently hydrolyze under the influence of internal material moisture or environmental humidity, forming carboxylic acids. Hydroxyl radicals generated by partial discharge can accelerate this process, further increasing the variety of carboxylic acid products.
(2) After discharge, the content of organic acids in the liquid products of epoxy resin is significantly higher than that of inorganic acids. PY-GC-MS and LC-MS/MS results indicate that the variety of organic acids generated by discharge in the presence of a specific atmosphere is greater than those produced solely by thermal effects. Moreover, the types of organic acids formed on the surface of the epoxy resin are more diverse than those within its interior. The greater variety and higher relative content of organic acids accelerate the surface corrosion and damage of ECR glass fibers, leading to the decay-like deterioration of composite insulators primarily occurring at the interface. Therefore, the impact of organic acids on glass fibers cannot be overlooked, and the study of this mechanism holds significant importance.
(3) The deterioration process of ECR glass fibers by organic acids involves multiple mechanisms. Firstly, oxalic acid degrades the coupling agent coating on the surface of the glass fibers, exposing their internal structure. Secondly, the H^+^ ions in oxalic acid react with the alkali metal–silica network within the glass fibers, leading to the dissolution and leaching of metal ions, thereby compromising the structural integrity of the fibers. Additionally, the leached metal ions combine with oxalate ions to form insoluble complexes, further accelerating the decay-like deterioration process.
(4) Elevated temperatures significantly accelerate the degradation process of ECR glass fibers. In practical applications, under the influence of external mechanical stress, surface cracks on the glass fibers gradually propagate, ultimately leading to their fracture. This indicates that environmental temperature, acidic solutions, and the duration of degradation are critical factors influencing the decay-like deterioration and even fracture of glass fibers.
(5) Under the same experimental temperature conditions, compared to nitric acid, the oxalic acid experimental group exhibited significant changes in mechanical properties, chemical elements, and functional groups, indicating that oxalic acid has a more pronounced corrosive effect on glass fibers.
(6) To enhance the service life of composite insulators, this study recommends optimizing production processes and standards before the products leave the factory, thereby reducing defects that lead to partial discharge. Additionally, during the production process, glass fiber materials with lower Ca content should be selected, and the formulation of the core rod should be adjusted to minimize the generation of organic acids.

## Figures and Tables

**Table 1:** Organic acidic products generated by simulating instantaneous thermal effect of discharge.

RT (min)	Name	Formula	Structure	Mol. wt.	pKa
12.25	cis-3-Methyl-4-cyclohexene-1,2-dicarboxylic acid	C_9_H_12_O_4_	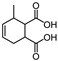	184	4.3
21.91	4’-Ethyl-4-biphenylcarboxylic acid	C_15_H_14_O_2_	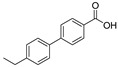	226	4.2
11.20	Benzoic acid, 3-methyl-	C_8_H_8_O_2_	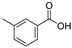	136	4.3
19.20	n-Hexadecanoic acid	C_16_H_32_O_2_	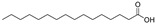	256	4.8
11.89	1,2-Benzenedicarboxylic acid	C_8_H_6_O_4_	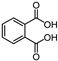	166	2.95

**Table 2:** Acidic products from discharge decomposition under the combined effect of thermal effects and environmental active species.

RT * (min)	Name	Formula	Structure	Mol. wt.	pKa	Area
(−)4.85	Ethylmalonic acid	C_5_H_8_O_4_	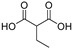	132.04	3.1	8.0 × 10^8^
(+)5.23	Adipic acid	C_6_H_10_O_4_	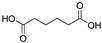	146.06	4.5	3.9 × 10^8^
(+)13.08	4-Methoxycinnamic acid	C_10_H_10_O_3_	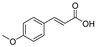	178.19	4.6	3.6 × 10^8^
(−)11.52	Hexadecanedioic acid	C_16_H_30_O_4_	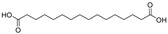	286.21	4.7	3.1 × 10^8^
(−)1.38	Acetic acid	C_2_H_4_O_2_	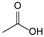	60.02	4.7	2.2 × 10^8^
(−)11.66	16-Hydroxyhexadecanoic acid	C_16_H_31_O_3_	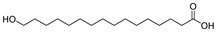	272.23	4.8	2.1 × 10^8^
(+)5.35	trans-3-Hexenoic acid	C_6_H_10_O_2_	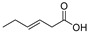	114.07	4.5	1.9 × 10^8^
(−)2.13	Nitric acid	HNO_3_	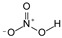	63	−1.4	1.8 × 10^8^
(−)6.94	Suberic acid	C_8_H_14_O_4_	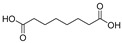	174.09	4.5	1.3 × 10^8^
(+)11.66	Palmitoleic acid	C_16_H_30_O_2_	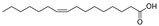	254.22	4.8	1.1 × 10^8^
(−)4.80	2-Methylglutaric acid	C_6_H_10_O_4_	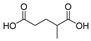	146.06	4.6	1.1 × 10^8^
(−)3.16	Methylsuccinic acid	C_5_H_8_O_4_	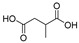	132.04	4.6	1.1 × 10^8^
(−)2.35	D-α-Hydroxyglutaric acid	C_5_H_8_O_5_	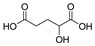	148.04	3.7	1.0 × 10^8^
(−)4.66	Citraconic acid	C_5_H_6_O_4_	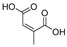	259.05	3.1	1.0 × 10^8^

* In the table, RT (+) and (−) represent the mass spectrometry data in positive and negative ion modes, respectively; the smaller the pKa value of an acidic substance, the stronger its acidity.

**Table 3:** Tensile breaking stress, modulus of elasticity, and reduction in both for glass fibers under different acid–heat conditions.

Deteriorating Conditions	Tensile Breaking Stress (MPa)	Decrease in Tensile Breaking Stress (%)	Elastic Modulus (GPa)	Decrease in Elastic Modulus (%)
Intact	529.20	/	55.72	/
25 °C-0.5 mol/L Oxalic acid-12 d	336.28	36.46	34.74	37.65
25 °C-1.0 mol/L Nitric acid-12 d	480.26	13.03	44.92	19.38
80 °C-0.5 mol/L Oxalic acid-12 d	20.51	96.12	3.22	94.22
80 °C-1.0 mol/L Nitric acid-12 d	311.34	41.17	37.26	33.13

**Table 4:** Reductions in different elements for glass fibers under different acid–heat conditions.

	Elements	C	Decrease in C (%)	O	Decrease in O (%)	Si	Decrease in Si (%)	Metal Elements	Decrease in Metal Elements (%)
Deterio- Rating Conditions									
Intact		23.35	/	29.87	/	23.35	/	23.43	/
25 °C-0.5 mol/L Oxalic acid-12 d		21.61	7.45	29.89	−0.07	26.35	−12.85	22.15	5.46
25 °C-1.0 mol/L Nitric acid-12 d		26.04	−11.52	27.2	8.94	25.5	−9.21	21.26	9.26
80 °C-0.5 mol/L Oxalic acid-12 d		15.18	34.99	41.93	−40.37	39.55	−69.38	2.94	87.45
80 °C-1.0 mol/L Nitric acid-12 d		15.18	34.99	38.59	−29.19	33.33	−42.74	12.9	44.94

The positive and negative values in the table indicate whether the content of the corresponding element decreases or increases under the given conditions compared to its intact state.

**Table 5:** Absorption peak areas of principal functional groups in glass fibers deteriorated by oxalic acid under various acid–heat conditions and their relative changes.

	Functional Group Content	Si-O	Si-O Content Relative to Intact Sample (%)	Si-O-Si	Si-O-Si Content Relative to Intact Sample (%)	-OH	-OH Content Relative to Intact Sample (%)
Deteriorating Conditions							
Intact		36.60	/	55.20	/	6.60	/
25 °C-0.5 mol/L Oxalic acid-12 d		30.40	83.06	44.44	80.51	8.10	122.73
25 °C-1.0 mol/L Nitric acid-12 d		31.13	85.05	45.82	83.01	6.60	99.97
80 °C-0.5 mol/L Oxalic acid-12 d		16.55	45.22	17.31	31.36	25.45	385.61
80 °C-1.0 mol/L Nitric acid-12 d		20.96	57.27	31.29	56.68	7.81	118.33

## Data Availability

The data presented in this study are available on request from the corresponding author. The data are not publicly available at this time due to the manufacturers’ data privacy requirements, as this work is conducted in collaboration with composite insulator manufacturers.

## Supplementary Materials

The following supporting information can be downloaded at: https://www.mdpi.com/article/10.3390/polym17111540/s1, Figure S1: TIC results supporting to choose acids shown in Table 1; Figure S2: Extracted-ion chromatogram (XIC) results of partial acid products worked out from Figure 9.

## Abbreviations

The following abbreviations are used in this manuscript:

FRP	Fiber-reinforced polymer
PY-GC-MS	Pyrolysis-gas chromatography-mass spectrometry
HPLC-MS-MS	High-performance liquid chromatography-tandem mass spectrometry
MeTHPA	Methyl tetrahydrophthalic anhydride
HESI	High-efficiency electrospray ionization source
