# Supplementary material for: Effects of Organic Acidic Products from Discharge-Induced Decomposition of the FRP Matrix on ECR Glass Fibers in Composite Insulators
Source: Polymers (Basel). 2025 May 31;17(11):1540. doi: 10.3390/polym17111540 (PMC12156956; doi:10.3390/polym17111540)
Supplement: Supplementary file 1 [file polymers-17-01540-s001.zip › polymers-3582298-supplementary.pdf]

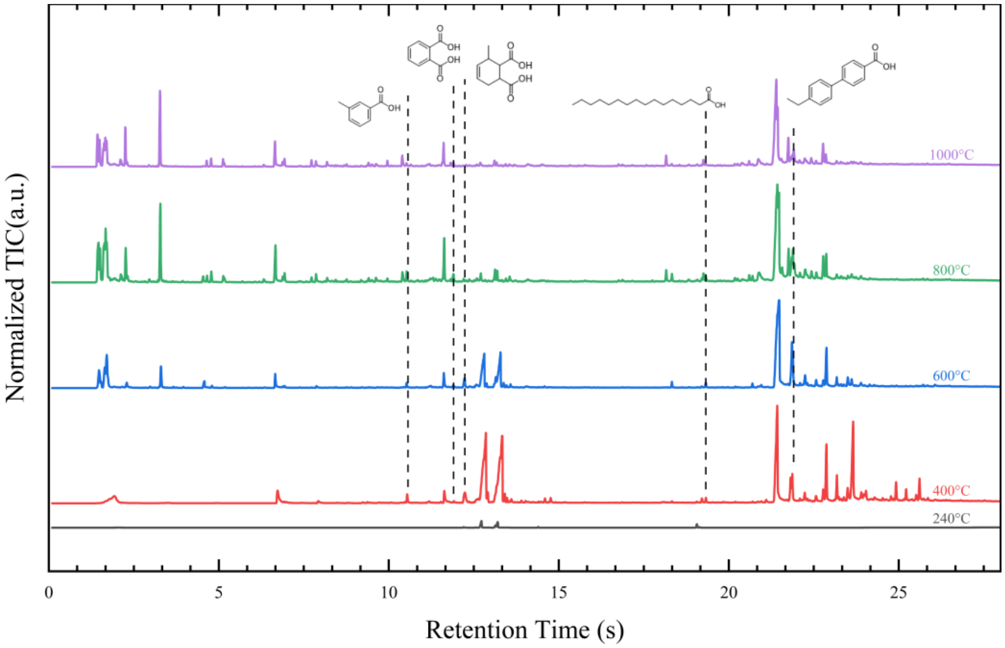

Figure S1 TIC results supporting to choose acids shown in Table 1.

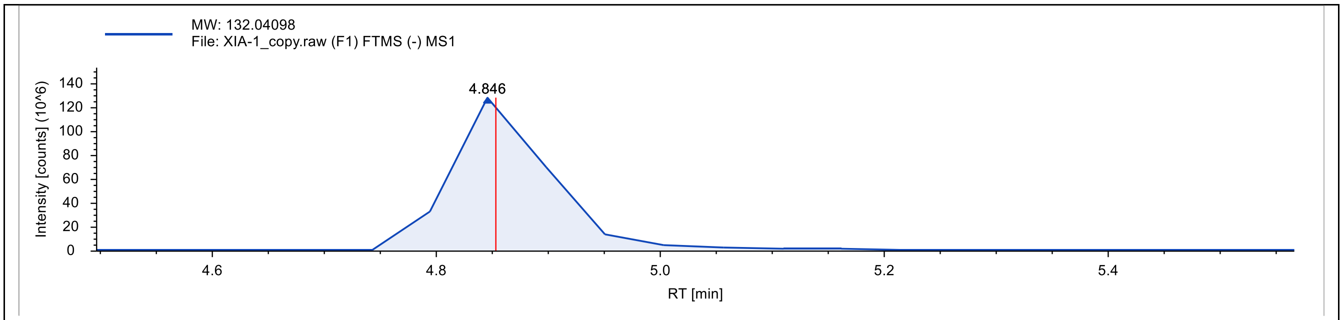

(a) Ethylmalonic acid at RT=4.85 min

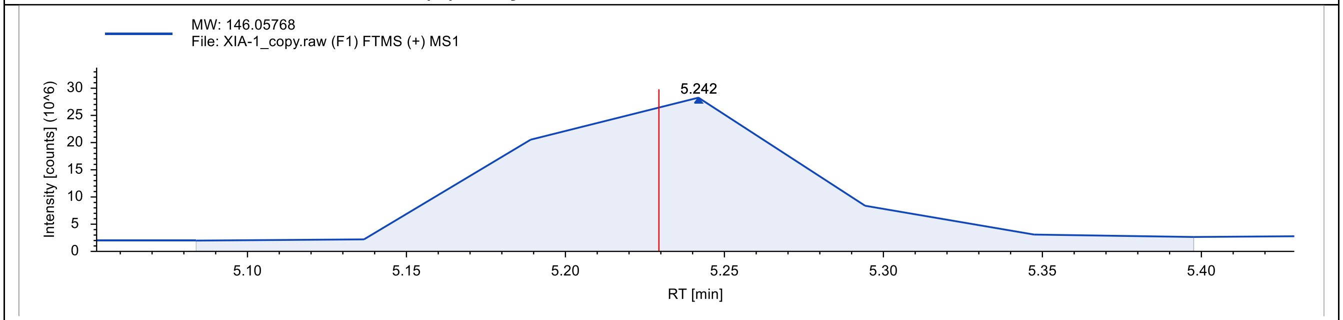

(b) Adipic acid at RT=5.23 min

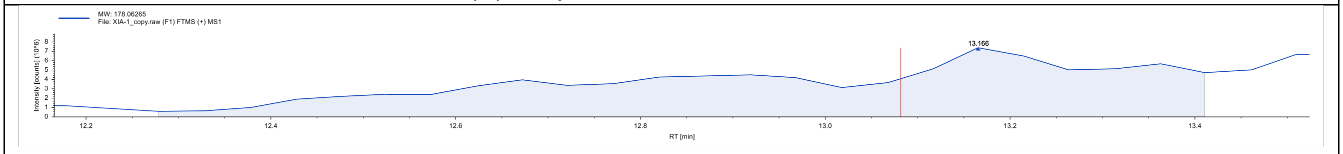

(c) 4-Methoxycinnamic acid at RT=13.08 min

Figure S2 XIC results of partial acid products worked out from Figure 9.
